# Correlations between Repeated Use of Dry Sauna for 4 x 10 Minutes, Physiological Parameters, Anthropometric Features, and Body Composition in Young Sedentary and Overweight Men: Health Implications

**DOI:** 10.1155/2019/7535140

**Published:** 2019-01-21

**Authors:** Robert Podstawski, Krzysztof Borysławski, Cain C. T. Clark, Dariusz Choszcz, Kevin J. Finn, Piotr Gronek

**Affiliations:** ^1^University of Warmia and Mazury in Olsztyn, Faculty of Environmental Sciences, Chair of Tourism, Recreation and Ecology, M. Oczapowskiego 5, 10-719 Olsztyn, Poland; ^2^Wrocław University of Life and Environmental Sciences, Department of Anthropology, Wrocław, Poland; ^3^Coventry University, Faculty of Health and Life Sciences, Coventry CV1 5FB, UK; ^4^Department of Heavy Duty Machines and Research Methodology, Faculty of Technical Sciences, University of Warmia and Mazury in Olsztyn, Oczapowskiego 11, 10-719 Olsztyn, Poland; ^5^University of Central Missouri, Department of Nutrition and Kinesiology, USA; ^6^University of Physical Education in Poznan, Department of Dance and Gymnastics, Poland

## Abstract

**Background:**

The effect of thermal stress on the physiological parameters of young overweight and sedentary men who sporadically use the sauna remains insufficiently investigated.

**Aim:**

The aim of the study was to determine the effect of sauna bathing on the physiological parameters of young overweight, physically inactive men and to test the correlations between physiological parameters versus anthropometric features and body composition parameters.

**Materials and Methods:**

Forty-five overweight and sedentary men aged 20.76±2.4 y were exposed to four sauna sessions of 10 minutes each (temperature: 90-91°C; relative humidity: 14-16 %) with four 5-minute cool-down breaks. Body composition was determined before sauna, and body mass and blood pressure were measured before and after sauna. Physiological parameters were monitored during four 10-minute sauna sessions.

**Results:**

A significant (p<0.0001) increase in all analyzed physiological parameters was observed during four successive 10-minute sauna sessions. Heart rate, energy expenditure, blood pressure, and body mass loss were most strongly correlated with anthropometric parameters (body mass, body mass index, and body surface area) and body composition parameters (percent body fat, body fat mass, and visceral fat level). The 60-minute treatment resulted in a significant reduction in body mass (0.65 kg).

**Conclusions:**

Repeated use of Finnish sauna induces significant changes in the physiological parameters of young sedentary overweight men, and these changes are intensified during successive treatments. Deleterious cardiovascular adaptations were most prevalent in men characterized by the highest degree of obesity and the largest body size.

## 1. Introduction

Sauna has emerged as a popular form of wellness treatment around the world in recent decades. Despite the above, many people visit saunas out of curiosity or the desire to follow the latest trends, and not all of them use saunas regularly [1]. Finland has a population of 5.2 million, and nearly 2 million Finns use saunas regularly, whilst other Scandinavians also take sauna baths at least once a week for health improvement [2, 3]. In addition, growing numbers of people install saunas at home or use different types of sauna (dry, steam, or infrared) in SPA centers [4]. Historically, saunas were popularized by Finnish athletes during the Olympic Games of 1936; consequently, sauna baths have been introduced to training programs in many sport disciplines [5].

Athletes and individuals purport using the sauna to cleanse the body, refresh the mind, and accelerate recovery and relaxation [6]. Regular sauna use improves adaptability to various environmental conditions, increases physical effort, and contributes to emotional wellbeing [7, 8]. In studies conducted by Scoon et al. [9] and Ernst et al. [10], postexercise sauna bathing over a period of three weeks substantially improved running performance, which can be attributed to an increase in blood volume. The Finnish sauna has been found to increase the endurance of locomotor and cardiorespiratory systems and physiological efficiency [7, 11]. In scuba divers, a single sauna session before diving has been shown to significantly decrease the number of circulating bubbles after a chamber dive, thereby minimizing the risk of decompression sickness [12].

Sauna baths are conducive to the treatment of locomotive organ inflammation, nonspecific ailments of the upper respiratory system [13–15], and sport-induced injuries [16–19]. Exercise can result in Exercise-Induced Muscle Damage (EIMD), which produces cramps, muscle strain, impairs muscle function, and delayed onset of muscle soreness [20]. In respondents who visited a sauna before EIMD, thermal treatment reduced sensory impairment (PF-ROM and PE-ROM) and improved muscle functions (GS and WES) in wrist extensor muscles [21]. Thermal treatment and rapid cooling after sauna were also found to exert a complex and positive effect on vascular and cardiac functions [4, 22], including arterial stiffness, BP, and some blood-based biomarkers [23].

Sauna treatment activates the endocrine system and promotes the secretion of epinephrine [2, 24–28], ACTH, cortisol, and prolactin as the body adapts to high temperature [29]. The endocrine system is stimulated to retain more water in the body and maintain thermal equilibrium [30]. Perspiration decreases serum sodium serum levels in the body [31]. Sauna bathing decreases total cholesterol levels and the concentrations of low-density lipids, and it increases the content of high-density lipids [32]. All of these responses might be viewed as beneficial for a person with chronic disease.

Far-infrared sauna improves the quality of life in people suffering from type 2 diabetes mellitus, chronic pain, chronic fatigue syndrome, depression, and congestive heart failure [33]. Finnish sauna, a thermal treatment that heats the entire body, has been found to produce positive clinical effects in rheumatism patients [34]. In rheumatism sufferers, regular sauna use reportedly alleviates pain associated with musculoskeletal injuries and improves joint mobility [2, 35]. The application of superficial heat is recommended as a short-term palliative treatment for rheumatoid arthritis and low back pain [36].

The uses of sauna treatment to facilitate health outcomes in persons with moderate risk of cardiovascular disease (i.e., sedentary behavior, overweight/obese, hypertension, and hyperlipidemia) have not been adequately studied. Beever [33] reviewed the literature using the terms “far-infrared” and “sauna” which yielded only nine useful studies. Among these studies, five supported the use of far-infrared sauna for reducing coronary heart disease risk factors. In addition, a recent investigation by Laukkanen et al. [37] showed a negative association between the frequency of sauna bathing and fatal CV events over a 20 year period suggesting a potential benefit from sauna treatments, but these investigators observed that further research was needed to establish a potential mechanism.

The influence of high temperature on physiological parameters and body fluid loss in individuals with different body size (body mass and height) has been weakly researched. Dry sauna leads to changes in physiological parameters as well as body composition. Sauna bathing induces sweating, which promotes passive dehydration and leads to hyperthermia [18], mainly due to the evaporation of sweat and enhanced blood circulation in the skin, the main cooling mechanism in the body [4]. Uncontrolled sweating leads to body mass loss (0.1-1.0 kg), and fluid loss can reach up to 13 liters under extreme circumstances, such as sauna competitions [38]. The body mass loss (BML) observed after sauna can be attributed mainly to the loss of body water. The above leads to changes in electrolyte levels, in particular sodium and chloride, subject to individual sweat rate and sweat composition [39]. Dehydration induced by severe sweating can compromise exercise performance and cognition [40–42]; therefore, during sauna treatment, visitors should minimize the risk of dehydration by matching their fluid intake with sweat loss.

A dehydration-related decrease in body mass has been empirically shown in humans [19]. Approximately 1 L of bodily fluids is lost through sweating, which corresponds to the loss of 1 kg of body mass [40, 41]. Sauna-induced body mass loss can be measured to determine the fluid intake that is required to compensate for that decrease. The physiological processes associated with sauna-induced thermal stress have been widely studied, but the mechanisms responsible for these processes have not yet been fully elucidated [2, 35]. The risks associated with excessive thermal stress in a sauna have been well documented, and dehydration, hyperthermia, and the resulting health problems can be prevented by monitoring BML. Dehydration combined with hyperthermia is far more dangerous for health than dehydration or hyperthermia alone [43].

The influence of thermal stress during prolonged sauna use on physiological parameters has limited research in conjunction with the correlations between somatic features and body composition. In addition, overweight and sedentary individuals have a potential additive thermal stress which might compromise the effects of sauna on health benefits. Therefore, the aim of this study was to determine the effect of thermal stress on physiological parameters and their correlations with somatic features and body composition parameters in young, overweight, and sedentary males.

## 2. Materials and Methods

### 2.1. Ethical Approval

The study was conducted upon the prior consent of the Ethics Committee of the University of Warmia and Mazury in Olsztyn (UWM), Poland. The study was performed on student volunteers who signed an informed consent statement.

### 2.2. Participants Selection

The study was conducted in 2017 on 45 full-time volunteer male students aged 19-24 years (20.76±2.4). The pool of potential participants were informed about the purpose of the study during obligatory physical education (PE) classes at the University of Warmia and Mazury in Olsztyn. The students who agreed to participate in the study (67 men) were notified by e-mail and text message whether they met the inclusion criteria and were provided with the date of final recruitment. Forty-five students meeting the below inclusion criteria were recruited for the study. The participants attended only mandatory PE classes (90 minutes per week); they did not undertake extracurricular physical activity and had rarely visited a sauna before the study. The participants confirmed that they did not take any medications or nutritional supplements, were in good health, and had no history of blood diseases or diseases affecting biochemical and biomechanical factors. None of the evaluated participants had respiratory or circulatory ailments. Physical activity (PA) levels (quantitative analysis) were evaluated with the use of the Polish short version of the standardized and validated International Physical Activity Questionnaire (IPAQ) [44]. The IPAQ was used only to select a homogenous sample of male students, and the results were presented only in terms of Metabolic Equivalent of Task (MET) units indicative of the participants' PA levels. The participants declared the average weekly number of minutes dedicated to PA (minimum of 10 minutes) before the study. The energy expenditure associated with weekly PA levels was expressed in terms of MET units [45]. The MET is the ratio of the work metabolic rate to the resting metabolic rate, and 1 MET denotes the amount of oxygen consumed in 1 minute, which is estimated at 3.5 mL/kg/min. Based on the declared frequency, intensity, and duration of PA, the respondents were classified into groups characterized by low L < 600 METs-min/week), moderate (M < 1,500 METs-min/week), and high (H ≥ 1,500 METs-min/week) levels of activity. Only male students characterized by low levels of PA (energy expenditure of up to 600 METs per week) and a sedentary lifestyle were chosen for the study.

### 2.3. Instruments and Procedures

The participants received comprehensive information about sauna rules during PE classes preceding the study. They were asked to drink at least 1 L of water on the day of the test and 0.5 L of water 2 hours before the session. The participants did not consume any foods or other fluids until after the final body measurements.

All participants visited a dry sauna during weekly PE classes on the same day, in the same location and over the same period of time, to minimize the effect of diurnal variation on the results [46]. Every participant attended four sauna sessions (temperature: 90°C; relative humidity: 14-16 %) of 10 minutes each and remained in a sitting position during each session. After every 10 minute session, students recovered in a room with a temperature of 18°C. Every recovery session lasted 5 minutes, during which the participants took a shower set to a temperature of 14-15°C. The volunteers could also cool down in a paddling pool (pool width: 100 cm; pool depth: 130 cm; water temperature: +10°C).

Body height was measured to the nearest 0.1 mm with a stadiometer, and nude body mass was measured to the nearest 0.1 kg with a calibrated WB-150 medical scale (ZPU Tryb Wag, Poland) prior to the first sauna session. Nude body mass was also measured after the last 5-minute cooling break (after 60 minutes of the experiment) to calculate body mass loss (BML). Somatic features, including body mass, body mass index (BMI), body surface area (BSA), and the waist-hip ratio (WHR), and body composition parameters, including body mass, total body water (TBW), protein and mineral content, body fat mass (BFM), fat-free mass (FFM), skeletal muscle mass (SMM), percent body fat (PBF), InBody score, target weight, visceral fat level (VFL), basal metabolic rate (BMR), and degree of obesity, were determined by bioelectrical impedance [47] with the InBody 720 body composition analyzer before the first sauna session. During exposure to high temperature in the sauna, physiological parameters, including heart rate (HR_min,avg,max_), recovery time, peak training effect (PTE), energy expenditure, estimated oxygen uptake (VO_2_  _avg,max_), estimated excess postexercise oxygen consumption (EPOC_avg,peak_), estimated respiratory rate (_avg,max_), and physical effort (easy, moderate, difficult, very difficult, maximal), were measured indirectly with Suunto Ambit3 Peak Sapphire heart rate monitors which are widely used in studies of the type [9]. HR monitors were placed on the left or right wrist (for left-handed and right-handed participants), and the sensors were attached to the chest. Every pulsometer was calibrated to male sex, year of birth, body mass, and PA level. Blood pressure (BP) was determined with an automatic digital blood pressure monitor (Omron M6 Comfort, Japan) before the first session and immediately after each session with the participant remaining in a sitting position.

### 2.4. Statistical Analysis

The measured data were processed statistically in the Statistica PL v. 10 application with the use of descriptive statistics. The values of the asymmetry coefficient (AC) were calculated to analyze the normality of distribution. The arithmetic means of the parameters measured after each of the four sauna sessions were compared by one-way (univariate) analysis of variance (ANOVA). The Least Significant Difference (LSD) post hoc test was performed when the F value was statistically significant. The above test is particularly recommended for planned repetitive experiments or longitudinal data with equal group size. The direction and strength of the relationships between interval features were determined by calculating Pearson's correlation coefficient (*r*).

## 3. Results

The results are presented in five tables. The participants' anthropometric features and physiological parameters are presented in Table 1.

The surveyed male students lost a significant (p<0.001, t=18.33, df=44) amount of bodily fluids (0.65±0.24 kg) after the 60-minute experiment (4 sauna sessions of 10 minutes each with four 5-minute breaks between sessions). The participants' average body mass (85.86 kg) was excessive relative to height (179.71 cm), and the subjects were classified as overweight based on their BMI (26.55 kg/m^2^) according to WHO standards. The lowest BMI values (18.75 kg^.^m^2^) were within the norm, whereas the highest BMI values (40.23 kg^.^m^2^) were indicative of class III obesity. The waist-hip ratio (0.89) approximated the upper limit of the healthy range (WHR>0.90) and was not indicative of android obesity (WHR ≥1), with relatively high values of VFL (7.56 kg) and high degree of obesity (120.96). According to the percent body fat scale developed by Thompson [48] and the noted BFM values (18.94 kg), the evaluated students (PBF=21.87%) belonged to a “potential risk” group (19.0-24.0%). High BFM values were accompanied by relatively high values of SMM and FMM (38.11 and 66.9 kg, respectively), whereas the average values of systolic (SBP) and diastolic blood pressure (DBP) were within the norm (125.84 mmHg and 81.38 mmHg, respectively).

The values of HR_min,mean,max_ increased significantly (p<0.001) during successive 10-minute sauna sessions with 5-minute breaks between sessions. The average HR was determined at 98 bpm during the first sauna session and at 133.36 bpm (difficult effort) during the fourth session. Maximum HR values (HR_max_) reached up to 160 bpm (maximal effort) during the fourth session. The recommended recovery time (calculated automatically by HR monitors) was 0.20 h after the first sauna session, and it differed significantly (p<0.001) after the fourth session at 2.69 h. The participants burned 73.04, 93.82, 114.91, and 131.40 kcal during every successive 10-minute session in the sauna. Between the first and the fourth session, the values of VO_2_  _mean_ and VO_2_  _max_ increased significantly from 11.44 (1^st^) to 26.44 L/min/kg (4^th^) and from 20.44 (1^st^) to 30.40 L/min/kg (4^th^), respectively. A significant (p<0.001) increase was also noted in EPOC_mean_ and EPOC_peak_ values (1.78-12.82 L/min and 3.93-25.31 L/min, respectively). The average respiratory rate was 17.18 breaths per minute during the first 10-minute session, and it increased significantly (p<0.001) to 23.44 breaths per minute during the fourth session. The values of SBP and DBP also increased significantly (p<0.001) from 128.40 (1^st^) to 140.67 mmHg (4^th^), and from 82.89 (1^st^) to 94.27 mmHg (4^th^), respectively. The highest number of HR readouts were within the easy effort range (<107 bpm: 464.1 s) during the first sauna session, within the moderate effort range (107-124 bpm: 312.7 and 265.8 s, respectively) during the second and third session, and within the difficult effort range (125-141 bpm: 416.7 s) during the fourth session. Between the second and fourth sauna session, HR values increased significantly (p<0.001) within the very difficult effort range (142-159 bpm; from 1.1 s to 99.0 s), and HR values indicative of maximal effort (≥160 bpm) were not observed (Table 2).

Body height was not significantly correlated with any of the analyzed body composition parameters, excluding DBP during the first sauna session (r=0.31). All HR values were significantly correlated with body mass, BMI, BSA, and WHR during the third and/or fourth session. Energy expenditure was significantly correlated with all body composition parameters between the second and the fourth session. The values of VO_2avg_ were bound by a significant positive correlation with BMI and WHR during the fourth session, whereas a significant negative correlation was noted between VO_2max_ and body mass, BSA, and WHR during the second session. The values of EPOC_avg, peak_ and respiratory rates_avg, max_ were significantly correlated with body mass, BMI, BSA, and WHR during the fourth session, and significant correlations were observed only incidentally in the remaining cases. Blood pressure (SBP and DBP) was most significantly correlated with body mass, BMI, BSA and WHR during all sauna sessions. The increasing values of correlation coefficients during every successive sauna session point to increasingly stronger correlations between the analyzed anthropometric features and indicators (mainly body mass, BMI, BSA, and WHR) and physiological parameters (HR_min, mean, max_, energy expenditure, VO_2_  _avg, max_, EPOC_avg, peak_, respiratory rate_avg, max_, and BP_SBP, DBP_) during prolonged sauna use (Table 3).

All analyzed values of HR_min, mean, max_ were significantly correlated with body composition parameters (TBW, proteins, minerals, BFM, FFM, SMM, and PBF) and PB_SBP, DBP_ values before sauna and during the third and fourth sauna session. Energy expenditure was significantly correlated with the above parameters already during the second session. Significant correlations between VO_2_  _avg, max_, EPOC_avg, peak_, and respiratory rate_avg, max_ were rarely observed, and they were noted mainly during the four session. The values of BP_SBP,DBP_ were most significantly correlated (between the first and the fourth session) with SBP and DBP before sauna. The increasing values of correlation coefficients during successive sauna sessions point to stronger correlations between body composition parameters (TBW, proteins, minerals, BFM, FFM, and SMM) and energy expenditure, and, to a smaller extent, between body composition parameters and HR, EPOC_avg, peak_, and respiratory rate_avg, max_ during prolonged sauna use. No such trends were observed in the correlations between SBP and DBP values during each session and before sauna, and the values of* r* were highly varied (Table 4).

The values of HR_min, mean, max_, EPOC_avg, peak_, and respiratory rate_avg, max_ were significantly correlated with body composition parameters (PBF, BFM, FFM, VFL, obesity degree) mostly during the fourth session and, less frequently, during the third session; and they were correlated with BML during the first and second session. Energy expenditure was significantly correlated with BML during every session, and with the remaining parameters during the second or the third session. The values of* r* decreased for the correlation with BML and increased for the correlations with the remaining parameters. The values of BP _SBP, DBP_ were most significantly correlated with PBF, BFM, WFL and obesity degree (increasing trend). Body mass loss was significantly correlated only with SBL (decreasing trend). The increasing values of correlation coefficients during successive sauna sessions point to stronger correlations between the analyzed body composition parameters (excluding BML) versus energy expenditure, BP_SBP, DBP_, and, to a lesser extent, between body composition parameters versus HR_min, mean, max_, EPOC_avg, peak_, and respiratory rate_avg, max_ during prolonged sauna use (Table 5).

## 4. Discussion

The current study produced a number of interesting observations. The energy expenditure of the evaluated males during four 10-minute sauna sessions was most frequently correlated with body mass, somatic indicators (BMI, BSA, and WHR), body composition parameters (TBW, proteins, minerals, BFM, FFM, SMM, BFM, VFL, and obesity degree), and BP_SBP, DBP_ before sauna use. These findings suggest that the energy expenditure of young men during sauna bathing is influenced by various factors, in particular body mass (39<* r* <70), BMI (37<* r* < 81) and BSA (37<* r* < 63). This study demonstrated that individuals with higher body mass, body area, body fat mass and muscle mass expend relatively more calories during sauna bathing. The presented results also confirmed previous findings that energy expenditure is influenced by the duration of sauna bathing. During the first 10 minutes, the evaluated males expended around 73 calories on average, but their energy expenditure increased significantly (p<0.001) to more than 134 calories during the last 10-minute session. In participants with the highest values of anthropometric features and body composition parameters, maximal energy expenditure reached 153 calories during 10 minutes of sauna use.

Increased energy expenditure was accompanied by growing values of HR_avg_ during successive sauna sessions (from 98 bpm during the first session to more than 133 bpm during the fourth session). These results indicate that average physical effort during the fourth sauna session was within the difficult range (125-141 bpm). The highest HR values within the very difficult range (142-159 bpm) were also noted during the fourth session, reaching up to 153 bpm in extreme cases. The above results were also observed in men with the highest values of somatic indicators. In young people who regularly use the sauna, HR increases to approximately 100-110 bpm and may exceed 140-150 bpm with a rise in ambient temperature [14, 24, 25, 49–53]. The increase in HR can be even higher in participants who do not use the sauna regularly, which can be attributed to the lack of physiological adaptation to high temperature [25]. The rise in HR is also influenced by other factors, such as the length of stay in the sauna, age, sex, and physical endurance [54]. In this study, male participants rarely used the sauna and were characterized by low PA and overweight; and in extreme cases, the noted HR_max_ values approximated the lower boundary of the maximal effort range (≤ 160 bpm).

In terms of health outcomes, the increase in HR to around 120 bpm is regarded as a beneficial adaptive response, whereas an increase in excess of 140 bpm can have adverse consequences because it is associated with higher cardiac effort and diastole shortening [54]. The mechanism underlying the rise in HR probably relies on an increase in blood temperature and reflex stimulation of energetic cardiac beta-receptors [50]. High humidity in the sauna room also influences HR, but humidity was relatively low (14-16%) in this study. Heart rate decreases slowly during prolonged and gradual body cooling, such as a cold shower. Baseline HR is restored approximately 1-4 hours after sauna if the body is cooled at room temperature [50] In the present study, all participants took a cold shower during 5-minute breaks, and only 9 men stepped into a cold paddling pool (11-12°C) for less than 20 seconds. The evaluated students spent the remainder of the break in a sitting position in a room with a temperature of 25°C. Despite the above, the participants' HR values did not return to baseline values, but continued to increase during successive sauna sessions. The above could suggest that 5-minute breaks were not long enough for participants whose HR and BP values exceeded the recommended norm during successive sauna sessions.

Sauna bathing at a temperature of 90°C can induce massive bodily effort which not always delivers positive health effects, both physiological and psychological. Similar observations were made in our previous study [1] which demonstrated that sauna bathing had a highly relaxing and calming effect on most students; notwithstanding, in Podstawski et al. [1], 23.7% men and 14.93% women experienced discomfort due to excessive temperature, claustrophobia, excessive number of sauna users and the presence of the opposite sex. The effect of sexual dimorphism seems to be more linked with the participants' sociocultural status, which was confirmed in our successive study [6]. The average values of HR_min_ during the first sauna session were relatively high (83.07 bpm), which could be indicative of psychological discomfort.

Similar values of HR (82.7 bpm) before sauna use were also observed in men who did not train professionally and visited a sauna sporadically. These results were significantly higher than in men with average and high training levels (71.8 bpm and 68 bpm, respectively) who were regular sauna users. During a 30-minute stay in the sauna (3 sessions of 10 minutes each with 5-minute breaks; temperature: 83°C; humidity: 12-14%), the HR values of sedentary men increased most significantly (119.9 bpm) relative to the participants with average and high training levels (107 and 83.6 bpm, respectively) [55]. In a study by Pilch et al. [11], the HR values of 10 professional swimmers and 10 untrained students (aged 20-23 years), participating in three 15-minute sauna sessions with 5-minute breaks (temperature: 92.3°C, humidity: 27.4%), increased from 74 and 108 bpm to 133 and 144 bpm, respectively, after the third session. In a follow-up study conducted on 10 males (aged 25-28 years), who attended three 15-minute sauna sessions with 5-minute breaks (temperature: 91°C, humidity: 5-18%), Pilch et al. [56] observed that HR values increased significantly from 66.6 bpm before sauna to 126 bpm immediately after sauna. A study of 8 healthy men (mean age of 30 years, in the range of 22 to 53 years) exposed to a temperature of 80-90°C for 30 minutes in a sauna revealed an increase in HR values from 78.5 to 103.6 bpm [57]. The HR values of scuba divers did not change significantly after a 30-minute session in a dry sauna (65°C) [12]. The HR values of professional runners were determined at around 140 bpm after 31 to 35 minutes of sauna bathing (temperature: 89.9±2.0°C, humidity: up to 18%) [9].

Blood pressure (SBP and DBP) increased significantly after successive sauna sessions, and the average BP during the fourth session (140/94 mmHg) was indicative of stage 1 hypertension [58], which could point to preexisting health conditions that were manifested under exposure to thermal stress. A study of men with various training levels (high, average and men who did not train professionally) revealed an increase in SBP values (from 125.7 to 133, from 121.5 to 129.6, from 113.4 to 119.4 mmHg) and a decrease in DBP values during sauna (from 73.47 to 69.4, from 75.6 to 73.5, from 71.4 to 70.1 mmHg, respectively) [55]. Three 15-minute sessions separated by 5-minute breaks (temperature: 92.3, humidity: up to 17.4%) increased SBP values from 122.6 to 142.6 mmHg and decreased DPB values from 78.7 to 63.7 mmHg in 10 healthy males aged 25-28 years [56]. After 30 minutes of bathing in a dry sauna (65°C), SBP values decreased significantly (112±10 mmHg, p=0.013), whereas DBP values remained unchanged [12]. In men aged 22-53 years exposed to a temperature of 80-90°C for 30 minutes in a sauna, SBP values increased from 118 to 120 mmHg [57]. In the present study, the highest BP of 158/121 mmHg was noted in participants with the highest values of somatic indicators and body composition parameters (overweight or obesity). The values of SBP and DBP are generally higher in overweight or obese men and women than in normal weight individuals [59]. These values are often indicative of hypertension and indeed other comorbidities [60]. For this reason, it is advocated that individuals with diagnosed hypertension should use the sauna at lower temperatures (45-50°C), which are characteristic of steam sauna, but are not accompanied by high humidity (100%) [17, 54]. Humidity is low in the Finnish sauna (5-10%), which stimulates hemodynamic changes, including a decrease in BP and vascular resistance [61]. Air humidity is increased by pouring water onto heated stones, which induces a minor and transient (3-15 mmHg) increase in SBP [50]. Imamura and Kihara demonstrated a minor, but statistically significant decrease in the BP values of patients who regularly visited an infrared sauna over a period of two weeks [13, 17]. In people who assume a seated position in the sauna, BP may not be maintained within a constant range because peripheral vasodilation in the lower limbs and the absence of muscle pump activity under exposure to high temperature can decrease reflex vasoconstriction and venous return [54, 62]. Coronary vasospasm can further disrupt the equilibrium between oxygen demand and oxygen supply to the myocardium [63], and it increases the risk of arrhythmia, myocardial ischemia and infarction [62, 64]. In this study, male students maintained a standardized sitting position in the sauna, which could increase their SPB and DBP, especially that the participants were overweight, had a sedentary lifestyle and rarely visited the sauna. The above risks are particularly high in people suffering from coronary atherosclerosis, which is why cooling by whole body immersion is not,* de facto*, recommended for every sauna user [65, 66]. Sauna bathing rarely confers undesirable effects on participants with a healthy cardiovascular system [62, 67]. Cooling by immersion in cold water is not recommended for patients with cardiovascular problems, however, gradual cooling, including in a shower, is advised [68].

The effects of sauna bathing on BP reported in the literature vary considerably, depending on the applied method of measurement, type of sauna, duration of exposure which elicits the evaporation effect, and user adaptation to high temperature. Considerable variations were reported in studies where BP was measured with a sphygmomanometer, ranging from a minor increase [2, 69] or the absence of any changes [52, 70–72] to a decrease in SBP [17, 72–74] and DBP values [13–15, 17, 25, 49, 50, 52, 75].

The body mass loss associated with sauna bathing is also a very important indicator. Sweat volume during sauna bathing is estimated at 0.6 to 1.0 kg/h, and sweating is generally intensified with a rise in temperature and humidity, although individual responses may vary [52]. During 40 minutes of sauna bathing, the BML of the evaluated males was estimated at 0.65 kg, and it accounted for 0.75% of their body mass. The values of BML were also significantly correlated with anthropogenic indicators, body composition parameters and physiological parameters. The results of our study indicate that persons with a high BMI are more prone to dehydration, which is why individuals should replenish lost fluids during sauna bathing. Analogous results were noted in our previous study [19] which demonstrated significant correlations between BMI and BML and highlighted that the loss of bodily fluids in a dry sauna can be accurately predicted based on BMI values. Body mass loss, expressed as a percentage of total body mass increased disproportionately with an increase in the subjects' BMI. Body mass loss was lowest in underweight students, and it was higher in participants with normal body weight. BML values were very high in overweight and obese males and females and were approximately twofold higher than in underweight women, with similar correlations were noted in men [19]. In our previous study [18], BML values measured after sauna bathing were significantly higher in female and male subjects with higher body mass, but they tended to be lower in taller participants (less so in men). With every kilogram increase in body mass, the corresponding BML values increased by 0.0144 kg in women and 0.0146 kg in men on average. In the present study, BML was not correlated with height, probably because this parameter was not correlated with physiological indicators such as HR, energy expenditure, VO_2_  _avg, max_, EPOC_avg, peak_, respiratory rate_avg, max_, and SBP (Table 3). Sweating begins shortly upon entering the sauna and peaks after approximately 15 minutes. The average total sweat secretion has been estimated at 0.5 kg [75]. Body core temperature increases by 0.1 to 0.25°C per every percent of BML [76, 77]. Kozłowski and Saltin [78] conducted one of the first studies into the effect of sweating on the fluid balance, analyzing sweating-induced dehydration in 6 healthy males who were exposed to a temperature of 80°C in a sauna for 2.5 hours, which is nearly four times longer than in our study. In Kozłowski and Saltin [78], the average BML during the 2.5-hour sauna session was determined at 3.1 kg (4.1%). In other studies, the average sauna-induced BML was estimated at 400–600 g [49, 79]. In a study of swimmers and untrained subjects who attended three sauna sessions of 15 minutes each (temperature: 92.3°C, humidity: up to 27.4%), BML was determined at 0.43 and 0.82 kg, respectively. The results of the present study were similar to those reported by Pilch et al. [54] who analyzed changes in BML and physiological and biochemical parameters in 10 healthy males attending three 15-minute sauna sessions (temperature: 91°C, humidity: 5-18%) with 5-minute breaks. The participants assumed a sitting position in the sauna and lost 0.72 kg of bodily fluids. Coles et al. [80] studied 10 male subjects who remained in a dry sauna for six 15-minute sessions (temperature 48.9°C) with 5-minute breaks between sessions. The experiment involved a euhydration trial and a dehydration trial. The participants did not ingest any fluids during the dehydration trial. The experimental procedure was identical in both trials, the only difference being that during the euhydration trial, the participants were asked to drink water in a volume corresponding to the amount of body mass lost in the previous sauna session. The subjects lost 0.33 kg of body mass (0.4%) in the euhydration trial and 1.99 kg (2.3%) in the dehydration trial. In a study of 30 nonobese and obese individuals exposed to a temperature of 80°C for three 20-minute sauna sessions, BML values (-1.82 kg; in the range of -1.53 to -2.04 kg; 3.0 ± 0.5%) differed significantly (p < 0.001) between nonobese (22.8 ± 1.6) and obese (28.5 ± 1.9) subjects [81]. Thomas et al. [82] determined the average BML (normalized to body mass %) of 12 healthy adults at 0.91 ± 0.34% (in the range of 0.33–1.4%) after a 30-minute session in the sauna. The body mass of scuba divers decreased significantly (-450±18 g; p<0.001) after 30 minutes in a dry sauna (65°C) [12].

## 5. Limitations

The use of Suunto Ambit3 Peak Sapphire heart rate monitors for measuring the participants' physiological parameters was a potential limitation of this study. However, the evaluated males were exposed to extreme temperature (90°C), and different measuring equipment could not have been used as effectively in a study conducted on a large and homogenous sample (45 males) with similar environmental conditions (day, hour, duration, temperature, and humidity). Future studies may therefore wish to examine the reliability and validity of various HR monitors in extreme conditions.

## 6. Conclusions

Young, overweight, and sedentary men (aged 19-24 years) who rarely use the sauna lose around 0.65 kg of bodily fluids on average during four 10-minute sauna sessions (temperature: 90°C, humidity: 15%) with four 5-minute breaks in between sessions (total of 60 minutes). The noted values of physiological parameters (energy expenditure, HR_min, avg, max_, BP_SBP, DBP_, VO_2  avg  and  max_, EPOC_avg  and  peak_, and respiratory rate_avg  and  max_) increased significantly after every 10-minute session and even exceed the recommended norms (HR and BP during the third and fourth session). In some cases, elevated BP values could be indicative of preexisting health conditions that were manifested upon relatively long (40 minutes) exposure to thermal stress. Sauna sessions lasting 40 minutes could be excessive and dangerous to the health of men who are considerably overweight. The above parameters and BML were highly correlated not only with anthropometric indicators (in particular body mass, BMI, BSA, and WHR), but also with body composition parameters, in particular those indicative of high body fat content (BFM, PBF, and VFM) and high degree of obesity. Physiological parameters are less correlated with TBW, minerals, proteins, SMM, and initial values of SBP and DBP. These correlations significantly contribute to the loss of bodily fluids. The results of this study complement previous research findings and expand our knowledge about factors which significantly affect physiological parameters during sauna bathing. As such, these findings should be acutely considered by individuals, practitioners, and clinicians in the adoption of sauna exposure.

## Figures and Tables

**Table 1:** Descriptive statistics of the studied anthropometric features and physiological parameters (N=45) before sauna.

**Features**	**Mean**	**SD**	**min-max**	**AC**
**Body mass [kg] before sauna**	85.86	14.89	55.90-137.70	1.33
**Body mass [kg] after sauna**	85.21	14.83	55.39-137.15	1.33
**Body mass loss [kg] ** **∗** **)**	0.65	0.24	0.34-1.23	-0.97
**Body height [cm]**	179.71	6.45	166.0-194.0	-0.10
**BMI (Body Mass Index) [kg/m** ^**2**^ **]**	26.55	4.06	18.75-40.23	0.70
**BSA (Body Surface Area) [m** ^**2**^ **]**	2.07	0.19	1.63-2.67	0.97
**WHR (Waist-Hip Ratio)**	0.89	0.09	0.75-1.20	0.98
**TBW (Total Body Water) [L]**	48.98	6.33	32.90-64.00	-0.07
**Proteins [kg]**	13.29	1.74	8.90-17.20	-0.09
**Minerals [kg]**	4.63	0.64	3.24-6.25	-0.08
**SMM (Skeletal Muscle Mass) [kg]**	38.11	5.26	24.70-50.00	-0.12
**SBP (Systolic Blood Pressure) [mmHg]**	125.84	9.47	106-140	-0.54
**DBP (Diastolic Blood Pressure) [mmHg]**	81.38	5.97	69-92	-0.24
**PBF (Percent Body Fat) [**%**]**	21.87	7.23	7.30-46.30	0.49
**BFM (Body Fat Mass) [kg]**	18.94	9.96	5.00-63.80	2.28
**FFM (Fat Free Mass) [kg]**	66.9	8.68	45.00-87.50	-0.07
**VFL (Visceral Fat Level) [kg]**	7.56	4.57	1.00-28.00	2.22
**Obesity Degree**	120.96	18.55	85-183	0.65
**InBody score**	78.42	10.30	39-95	-1.01
**Target weight**	79.10	8.97	63-103	0.34
**BMR (metabolism) [kcal]**	1815.3	187.3	1342-2260	-0.07
**MET (Metabolic Equivalent of Task)** [**mL/kg/min]**	514.87	74.90	390-599	-0.51

***Key:*** AC: asymmetry coefficient, *∗t*= 18.33, *df*=44, and *p*<0.001.

**Table 2:** Comparison of the arithmetic means of physiological parameters (N = 45) after every sauna session [differences in arithmetic means are highly statistically significant *∗*)-*post hoc* LSD test].

**Parameter**	**Sauna session**												**Difference**	
	**1**			**2**			**3**			**4**				
	**Mean**	**SD**	**min-max**	**Mean**	**SD**	**min-max**	**Mean**	**SD**	**min-max**	**Mean**	**SD**	**min-** **max**	***F***	***p***
**H** **R**_**min**_ ** [bpm]**	83.07	8.30	67-103	88.96	8.07	71-103	98.84	9.36	72-114	108.18	11.38	80-128	62.74	<0.001
**H** **R**_**avg**_ ** [bpm]**	98.00	7.41	84-114	109.67	6.44	97-121	121.36	5.63	102-131	133.36	5.54	116-145	261.92	<0.001
**H** **R**_**max**_ ** [bpm]**	115.51	9.44	92-131	129.16	8.30	110-150	135.73	6.03	122-147	144.82	5.80	135-168	120.05	<0.001
**Recovery time [h]**	0.20	0.40	0-1	0.84	0.47	0-2	1.71	0.51	1-3	2.69	0.51	1-3	231.04	<0.001
**PTE-Peak Training Effect**	1.22	0.13	1.0-1.7	1.51	0.21	1.2-2.0	1.90	0.18	1.4-2.2	2.26	0.19	2.0-2.7	282.74	<0.001
**Energy expenditure [kcal]**	73.04	12.36	52-94	93.82	10.91	74-109	114.91	8.95	91-134	131.40	10.85	109-153	246.40	<0.001
**V** **O**_2**avg**_ ** [mL/kg/min]**	14.44	2.41	10-20	18.24	2.49	15-23	22.16	2.60	16-26	26.44	2.50	21-31	191.22	<0.001
**V** **O**_2**max**_ ** [mL/kg/min]**	20.44	3.73	13-27	24.82	3.54	18-33	26.93	3.22	19-32	30.40	2.73	24-35	70.49	<0.001
**E** **P** **O** **C**_**avg**_ ** [mL/kg]**	1.78	0.70	1-4	3.27	1.59	1-7	6.53	2.51	2-13	12.89	3.76	5-20	186.67	<0.001
**E** **P** **O** **C**_**max**_ ** [mL/kg]**	3.93	1.47	2-8	7.69	2.62	4-14	13.58	3.92	7-22	25.31	6.71	14-39	226.34	<0.001
**Respiratory ** **r** **a** **t** **e**_**avg**_ ** [brpm]**	17.18	1.35	15-20	18.93	1.63	15-23	20.87	1.47	18-25	23.44	1.85	20-27	128.96	<0.001
**Respiratory ** **r** **a** **t** **e**_**max**_ ** [brpm]**	22.76	1.36	20-25	24.89	1.68	22-29	26.24	1.60	24-31	29.62	2.72	25-37	102.06	<0.001
**Systolic blood pressure-SBP [mmHg]**	128.40	8.74	110-145	132.02	8.04	115-147	135.51	8.01	119-151	140.67	7.55	126-158	18.74	<0.001
**Diastolic blood pressure-DBP [mmHg]**	82.89	6.55	72-96	85.31	6.07	73-103	88.93	7.12	75-110	94.27	9.00	80-121	20.82	<0.001

**Physical effort**														

**Easy **<**107 [bpm]**	464.1	125.6	152-600	233.2	161.7	7-493	68.0	105.9	0-397	4.5	11.5	0-63	142.2	<0.001
**Moderate 107-124 [bpm]**	133.1	124.6	0-447	312.7	163.1	57-573	265.8	131.0	55-557	79.7	100.1	2-484	31.1	<0.001
**Difficult 125-141 [bpm]**	2.8	10.2	0-62	53.0	65.5	0-279	263.5	157.4	0-540	416.7	134.2	53-592	140.5	<0.001
**Very Difficult 142-159 [bpm]**	0.0	0.0	0-0	1.1	5.6	0-36	2.7	11.4	0-73	99.0	127.6	0-456	26.2	<0.001
**Maximal **≥** 160 [bpm]**	**All values are zero**													

*∗* indicates that the difference between the first and the second sauna session was not significant only for DBP (close to significance, *p *= 0.115).

**Table 3:** Correlations between anthropometric features and physiological parameters after successive sauna sessions (values in bold are statistically significant).

**Traits**	**Sauna session**	**HR**				**Energy expend. [kcal]**	**VO**_**2**_		**EPOC**		**Respiratory rate**		**Blood pressure**	
		**min**	**avg**	**max**	**max – min**		**avg**	**max**	**avg**	**peak**	**avg**	**max**	**SBP**	**DBP**
**Body height [cm]**	1	-0.05	-0.08	-0.07	-0.03	-0.17	-0.04	-0.06	-0.14	-0.07	-0.10	-0.15	-0.24	**-0.31**
	2	0.12	-0.12	-0.12	-0.17	0.11	-0.08	-0.19	-0.25	-0.05	0.02	0.12	-0.22	-0.27
	3	0.13	0.14	0.18	-0.01	0.15	0.01	0.00	-0.06	-0.9	-0.13	-0.09	-0.14	-0.23
	4	-0.13	-0.16	-0.26	0.00	-0.08	-0.23	-0.19	-0.22	-0.22	-0.20	-0.15	-0.16	-0.29

**Body mass [kg]**	1	-0.10	-0.11	-0.08	0.01	-0.04	-0.01	0.00	-0.09	-0.05	-0.10	-0.09	**0.53**	**0.32**
	2	0.25	0.07	-0.12	-0.26	**0.39**	-0.13	**-0.35**	-0.07	-0.09	0.08	-0.01	**0.52**	**0.36**
	3	0.21	**0.34**	0.28	-0.02	**0.66**	0.01	-0.05	0.10	0.23	0.14	0.18	**0.61**	**0.49**
	4	**0.36**	**0.44**	**0.31**	-0.22	**0.70**	0.19	0.06	**038**	**0.44**	**0.33**	**0.40**	**0.63**	**0.49**

**BMI (Body Mass Index) [kg/m** ^**2**^ **]**	1	-0.08	-0.07	-0.05	0.01	0.04	0.02	0.03	-0.02	-0.02	-0.05	-0.01	**0.71**	**0.50**
	2	0.23	0.13	-0.07	-0.21	**0.37**	-0.09	-0.28	0.05	-0.07	0.08	-0.07	**0.69**	**0.53**
	3	0.20	**0.33**	0.25	-0.04	**0.66**	0.03	-0.03	0.17	0.24	0.24	0.26	**0.75**	**0.66**
	4	**0.45**	**0.56**	**0.47**	-0.05	**0.81**	**0.31**	0.16	**0.51**	**0.59**	**0.45**	**0.51**	**0.77**	**0.68**

**BSA (Body Surface Area) [m** ^**2**^ **]**	1	-0.09	-0.11	-0.08	-0.00	-0.06	-0.00	0.00	-0.10	-0.06	-0.10	-0.10	**0.46**	0.24
	2	0.25	0.04	-0.13	-0.27	**0.37**	-0.13	**-0.35**	-0.11	-0.09	0.06	0.00	**0.45**	0.29
	3	0.22	**0.34**	**0.30**	-0.02	**0.64**	0.02	-0.03	0.09	0.24	0.11	0.15	**0.54**	**0.41**
	4	**0.32**	**0.37**	0.23	-0.22	**0.63**	0.12	0.01	**0.30**	**0.36**	0.26	**0.33**	**0.55**	**0.39**

**WHR (Waist-Hip Ratio)**	1	-0.23	-0.13	-0.01	0.17	-0.05	-0.08	0.03	-0.02	-0.03	-0.12	-0.17	**0.70**	**0.51**
	2	0.25	0.15	-0.05	-0.21	**0.35**	-0.11	**-0.31**	0.03	-0.07	0.13	-0.08	**0.71**	**0.59**
	3	0.06	0.09	0.27	0.11	**0.47**	-0.14	-0.02	-0.01	0.08	0.09	0.21	**0.72**	**0.61**
	4	**0.31**	**0.48**	**0.42**	-0.11	**0.54**	**0.33**	0.24	**0.47**	**0.55**	**0.31**	**0.40**	**0.79**	**0.68**

**Table 4:** Correlations between body composition parameters and blood pressure before sauna and physiological parameters after successive sauna sessions (values in bold are statistically significant).

**Parameters**	**Order of entry**	**HR**				**Energy expend. [kcal]**	**VO**_**2**_		**EPOC**		**Respiratory rate**		**Blood pressure**	
		**min**	**avg**	**max**	**max – min**		**avg**	**max**	**avg**	**peak**	**avg**	**max**	**SBP**	**DBP**
**TBW (Total Body Water) [L]**	1	0.14	0.07	0.02	-0.08	0.08	0.19	0.11	0.03	0.04	0.12	0.08	0.22	0.03
	2	0.22	0.03	-0.12	-0.23	**0.29**	-0.04	-0.22	-0.08	-0.03	0.01	0.04	0.17	0.04
	3	0.21	**0.37**	**0.33**	-0.00	**0.53**	0.12	0.04	0.09	**0.29**	0.08	0.11	0.27	0.18
	4	**0.29**	0.26	0.09	-0.27	**0.53**	0.01	-0.09	0.20	0.23	0.23	0.25	0.23	0.11

**Proteins ** **[kg]**	1	0.15	0.09	0.03	-0.09	0.11	0.21	0.12	0.05	0.05	0.14	0.10	0.25	0.06
	2	0.22	0.05	-0.10	-0.22	**0.30**	-0.02	-0.21	-0.07	-0.01	0.02	0.05	0.19	0.06
	3	0.21	**0.37**	**0.33**	0.01	**0.54**	0.13	0.05	0.10	**0.30**	0.08	0.12	**0.29**	0.19
	4	**0.29**	0.28	0.11	-0.26	**0.55**	0.02	-0.09	0.22	0.25	0.24	0.26	0.24	0.12

**Minerals [kg]**	1	0.08	0.02	-0.02	-0.07	0.06	0.14	0.07	-0.04	0.02	0.05	0.04	0.16	-0.02
	2	0.17	0.02	-0.10	-0.19	**0.30**	-0.02	-0.21	-0.09	0.01	0.00	0.08	0.13	0.02
	3	0.20	**0.38**	0.25	-0.04	**0.52**	0.10	-0.02	0.11	0.26	0.08	0.08	0.23	0.14
	4	**0.31**	0.22	0.06	**-0.31**	**0.49**	-0.03	-0.12	0.16	0.17	0.18	0.20	0.20	0.07

**SMM ** **(Skeletal Muscle Mass)** **[kg]**	1	0.15	0.08	0.03	-0.09	0.10	0.20	0.11	0.05	0.05	0.13	0.09	0.24	0.05
	2	0.22	0.04	-0.11	-0.23	**0.29**	-0.03	-0.22	-0.07	-0.02	0.02	0.04	0.18	0.05
	3	0.21	**0.37**	**0.33**	0.00	**0.53**	0.12	0.04	0.09	**0.29**	0.09	0.12	0.28	0.18
	4	**0.29**	0.27	0.11	-0.27	**0.55**	0.01	-0.09	0.21	0.24	0.23	0.25	0.24	0.12

**SBP (Systolic Blood Pressure) before sauna** ** [mmHg]**	1	-0.01	-0.01	-0.02	-0.00	0.15	0.03	0.03	0.06	0.01	-0.07	-0.11	**0.91**	**0.63**
	2	0.13	0.13	-0.05	-0.12	**0.36**	-0.10	-0.28	0.08	-0.09	0.03	-0.21	**0.90**	**0.66**
	3	0.13	0.24	0.26	0.04	**0.61**	0.04	0.07	0.14	0.20	0.17	0.27	**0.91**	**0.73**
	4	**0.48**	**0.46**	**0.43**	**-0.29**	**0.65**	0.24	0.15	**0.48**	**0.50**	**0.29**	**0.36**	**0.91**	**0.73**

**DBP (Diastolic Blood Pressure) before sauna** ** [mmHg]**	1	-0.08	0.01	0.10	0.15	0.11	0.04	0.14	0.13	0.12	0.03	0.04	**0.84**	**0.82**
	2	0.10	0.13	-0.05	-0.10	**0.32**	-0.05	-0.23	0.09	-0.04	0.09	-0.17	**0.80**	**0.85**
	3	0.13	0.23	0.26	0.04	**0.48**	0.10	0.08	0.19	0.20	0.20	0.28	**0.77**	**0.81**
	4	**0.36**	**0.42**	**0.36**	-0.20	**0.58**	0.22	0.13	**0.47**	**0.50**	**0.34**	**0.42**	**0.84**	**0.79**

**Table 5:** Correlations between **body composition parameters relating to adipose tissue **and physiological parameters after successive sauna sessions (values in bold are statistically significant).

**Parameters**	**Sauna session**	**HR**				**Energy expend. [kcal]**	**VO**_**2**_		**EPOC**		**Respiratory rate**		**Blood pressure**	
		**min**	**avg**	**max**	**max – min**		**avg**	**max**	**avg**	**peak**	**avg**	**max**	**SBP**	**DBP**
**PBF (Percent Body Fat) [**%**]**	1	-0.27	-0.21	-0.12	0.09	-0.07	-0.15	-0.06	-0.15	-0.10	-0.22	-0.17	**0.66**	**0.51**
	2	0.13	0.07	-0.08	-0.14	0.26	-0.15	**-0.29**	0.02	-0.10	0.03	-0.13	**0.69**	**0.55**
	3	0.09	0.15	0.19	0.03	**0.44**	-0.04	0.02	0.12	0.15	0.18	0.23	**0.69**	**0.60**
	4	0.26	**0.36**	**0.37**	-0.08	**0.52**	0.23	0.14	**0.34**	**0.42**	0.20	**0.30**	**0.77**	**0.66**

**BFM (Body Fat Mass) [kg]**	1	-0.27	-0.23	-0.14	0.09	-0.13	-0.18	-0.10	-0.16	-0.11	-0.25	-0.20	**0.61**	**0.44**
	2	0.19	0.08	-0.08	-0.19	**0.32**	-0.16	**-0.32**	-0.04	-0.11	0.11	-0.05	**0.64**	**0.51**
	3	0.13	0.18	0.14	-0.03	**0.53**	-0.10	-0.10	0.07	0.09	0.15	0.17	**0.67**	**0.58**
	4	0.28	**0.42**	**0.38**	-0.09	**0.59**	0.27	0.17	**0.38**	**0.46**	**0.30**	**0.38**	**0.74**	**0.64**

**FFM (Fat Free Mass) [kg]**	1	0.14	0.07	0.02	-0.08	0.08	0.19	0.11	0.03	0.04	0.11	0.08	0.22	0.04
	2	0.22	0.03	-0.11	-0.23	0.29	-0.03	-0.22	-0.08	-0.02	0.01	0.04	0.17	0.04
	3	0.21	**0.37**	**0.33**	-0.00	**0.53**	0.12	0.04	0.09	**0.29**	0.08	0.11	0.27	0.18
	4	**0.29**	0.26	0.09	-0.27	**0.54**	0.01	-0.09	0.20	0.23	0.23	0.25	0.23	0.11

**VFL (Visceral Fat Level) [kg]**	1	-0.27	-0.21	-0.11	0.11	-0.12	-0.16	-0.06	-0.14	-0.09	-0.22	-0.19	**0.62**	**0.45**
	2	0.21	0.10	-0.09	-0.21	**0.34**	-0.14	**-0.34**	-0.03	-0.10	0.11	-0.06	**0.64**	**0.51**
	3	0.11	0.19	0.16	-0.01	**0.53**	-0.09	-0.10	0.07	0.11	0.15	0.18	**0.67**	**0.58**
	4	**0.31**	**0.44**	**0.37**	-0.13	**0.59**	0.28	0.18	**0.41**	**0.48**	**0.31**	**0.39**	**0.74**	**0.64**

**Obesity Degree**	1	-0.06	-0.07	-0.06	-0.01	0.05	0.03	0.03	-0.02	-0.02	-0.05	-0.00	**0.71**	**0.48**
	2	0.22	0.11	-0.07	-0.20	**0.35**	-0.11	-0.28	0.04	-0.09	0.07	-0.08	**0.69**	**0.52**
	3	0.19	**0.33**	0.23	-0.04	**0.68**	0.03	-0.04	0.17	0.23	0.23	0.24	**0.75**	**0.65**
	4	**0.46**	**0.56**	**0.48**	-0.24	**0.82**	**0.31**	0.16	**0.52**	**0.59**	**0.46**	**0.50**	**0.77**	**0.68**

**BML (Body Mass Loss) [kg]**	1	**0.40**	**0.41**	0.20	-0.13	**0.59**	**0.32**	-0.10	0.25	**0.29**	0.07	-0.11	**0.36**	0.25
	2	0.14	**0.42**	0.03	-0.08	**0.55**	0.28	0.08	**0.34**	0.27	**0.33**	0.07	**0.35**	0.21
	3	0.02	0.14	0.20	0.10	**0.43**	0.04	0.03	0.11	0.08	0.20	**0.29**	**0.35**	0.20
	4	0.12	0.26	0.01	-0.12	**0.29**	0.08	-0.13	0.20	0.11	0.10	0.06	**0.33**	0.25

## Data Availability

The Excel data used to support the findings of this study are restricted by the Ethics Committee of the University of Warmia and Mazury in Olsztyn (UWM), Poland, in order to protect participants' privacy. Data are available from Robert Podstawski, e-mail: podstawskirobert@gmail.com, for researchers who meet the criteria for access to confidential data.
